# Enantiomeric Recognition of α-Aminoacids by a Uranyl Salen-Bis-Porphyrin Complex

**DOI:** 10.3389/fchem.2019.00836

**Published:** 2019-12-03

**Authors:** Chiara M. A. Gangemi, Ugne Rimkaite, Federica Cipria, Giuseppe Trusso Sfrazzetto, Andrea Pappalardo

**Affiliations:** ^1^Department of Chemical Sciences, University of Catania, Catania, Italy; ^2^Faculty of Chemistry and Geosciences, University of Vilnius, Vilnius, Lithuania; ^3^I.N.S.T.M. - Consorzio Interuniversitario Nazionale per la Scienza e Tecnologia dei Materiali, University of Catania, Catania, Italy

**Keywords:** porphyrins, salen ligands, uranyl complexes, luminescence, enantiomeric recognition

## Abstract

A novel uranyl salen-bis-porphyrin complex, in which two porphyrin subunits and salen moiety were directly linked, was synthesized for the recognition of tetrabutylammonium (TBA) amino acids. This uranyl salen complex, due to the presence of porphyrins with their fluorescence properties, represents the first example of a luminescence of uranyl salen complexes. UV/Vis measurements indicate the formation of 1:1 host–guest complexes, whereas UV-vis and fluorescence studies revealed that this complex acts as a receptor for the enantiomeric recognition of α-aminoacids derivatives, with high association constants and an excellent enantiomeric discrimination between the two enantiomers of phenylalanine–TBA.

## Introduction

Salen ligands are a class of molecules that have been widely explored in the field of supramolecular chemistry. The most fascinating and promising use of salen derivatives is due to their chiral complexes with numerous metals. The salen structure, due to two contiguous stereogenic carbon atoms in the diimine bridge, creates a chiral pocket which can coordinate a metal cation (*via* imine nitrogen and phenolic oxygen atoms). Salen ligands rose to prominence thanks to the pioneering work of Jacobsen and Katsuki that paved the way to one of the most well-designed protocol for the enantioselective epoxidation of unfunctionalized alkenes catalyzed by chiral manganese salen complexes (Jacobsen, 1993; Katsuki, 2000; Yoon and Jacobsen, 2003; La Paglia Fragola et al., 2012; Trusso Sfrazzetto et al., 2015; Ballistreri et al., 2016, 2018; Zammataro et al., 2019). Furthermore, salen ligands are structures of great value in homogeneous catalysis (Katsuki, 1995; Jacobsen, 2000; Cozzi, 2004; McGarrigle and Gilheany, 2005; Baleizao and Garcia, 2006; Wezenberg and Kleij, 2008; Whiteoak et al., 2012).

In recent years, our research group exploited these chiral salen-metal complexes as enantiomeric receptors for chiral guests. In fact, depending on the metal ion and different substituents in the aromatic ring of salen framework, these salen-metal complexes can be used as efficient enantioselective catalysts and highly sensitive chemosensors (D'Urso et al., 2014; Puglisi et al., 2017, 2018, 2019). In particular, chiral uranyl salen complexes have proved to be excellent receptors for amino acid salts (Amato et al., 2007, 2010, 2011; Ballistreri et al., 2010; Pappalardo et al., 2012a), since the uranyl metal center, acting as a Lewis acid, possesses an equatorial fifth position able to coordinate one molecule of carboxylate anion (Ballistreri et al., 2012; Brancatelli et al., 2013). These synthetic enantioselective receptors could help to better understand the mechanisms of drugs action; processes that are involved in immunological responses and processes of the storage of genetic information. Besides, slightly modification of their structures could lead to chemosensors, which due to their simple use, relatively low cost and high sensitivity are particularly significant in the chemical analysis.

**Figure 1:**
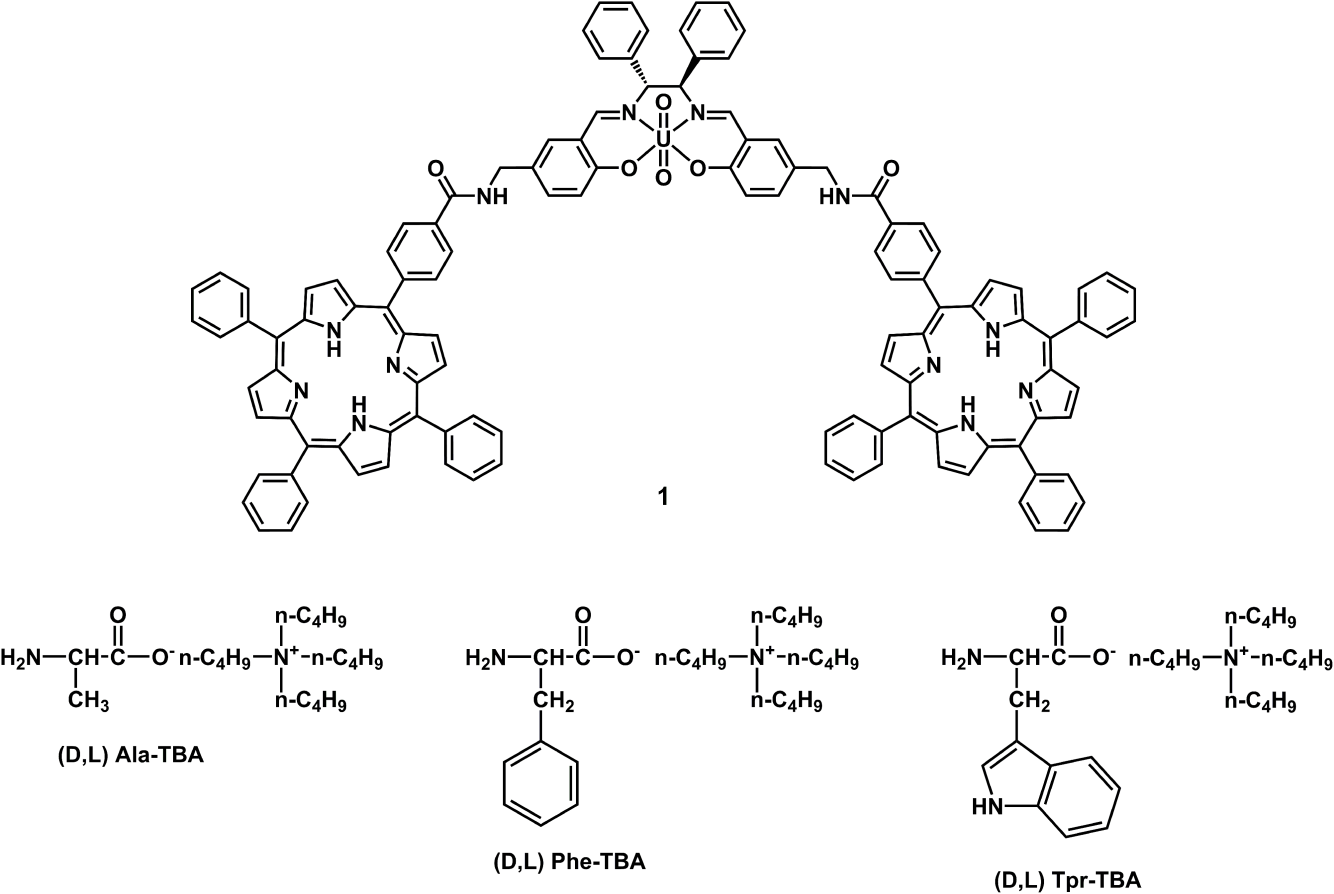
Uranyl salen-bis-porphyrin complex **1** and amino acid salts used as guests.

Taking into account the salen ligand applications in the field of catalysis and enantiomeric recognition, previous studies have inspired us to extend current research on the synthesis of salen receptors comprising porphyrin macrocycles which, with their high stability and fluorescence properties, could greatly extend the use of salen ligands as chemosensors. Porphyrins, due to their rigid molecular structure, tunable substituents, large skeleton dimensions, and additional metallation sites in the core, are very attractive macrocycles for their applications in many technological fields (Beletskaya et al., 2009; Drain et al., 2009). In our strategy, the rational combination of porphyrin derivatives with chiral uranyl-salen ligands in one structure, would lead to chemosensors that possess unprecedented luminescence properties, that till now were precluded in uranyl-salen complexes due to the presence of uranyl metal center. Here we report on the synthesis of a novel uranyl salen-bis-porphyrin complex, in which two porphyrin subunits and the salen ligand are directly connected, and the enantiomeric recognition properties of this receptor toward selected α-aminoacids derivatives assessed by UV-vis and fluorescence measurements (Figure 1).

## Materials and Methods

### General Experimental Methods

The NMR experiments were carried out at 27°C on a Varian UNITY Inova 500 MHz spectrometer (^1^H at 499.88 MHz, ^13^C NMR at 125.7 MHz) equipped with pulse field gradient module (Z axis) and a tuneable 5 mm Varian inverse detection probe (ID-PFG). ESI mass spectra were acquired on a API 2000– ABSciex using CH_3_OH (positive ion mode). A JASCO V-560 UV-Vis spectrophotometer equipped with a 1 cm path-length cell was used for the UV-Vis measurements. Luminescence measurements were carried out using a Cary Eclipse Fluorescence spectrophotometer with resolution of 0.5 nm, at room temperature. The emission was recorded at 90° with respect to the exciting line beam using 10:10 slit-widths for all measurements. All chemicals were reagent grade and were used without further purification.

### General Procedure for the Synthesis of TBA Amino Acid Derivates (Ballistreri et al., 2010)

An aqueous solution of tetrabutylammonium hydroxide (40% w/w, 13 mmol) was added to an aqueous suspension of the desired amino acid (13 mmol). The resultant reaction mixture was heated at 60°C for 2 h. Water was removed in vacuo at 80°C. The residue was dissolved in CH_2_Cl_2_ (10 mL), filtered and the solvent was evaporated in vacuo to afford in high yield the desired product.

### Procedure for UV-vis and Fluorescence Titrations

Two stock solutions of host and guest (1.0 × 10^−3^ M) in dry chloroform were prepared. From these, different solutions with different ratio receptor/guest (host concentration = 1.0 × 10^−6^ M) were prepared, and UV-vis and emission spectra were recorded at 25°C. Fluorescence titrations were carried out using λ_ex_ = 350 nm in dry chloroform, recording at λ_em_ = 650 and 715 nm. With this data treatment, the apparent binding affinities of receptor with amino acid guests were estimated using HypSpec (version 1.1.33) (Pappalardo et al., 2012b), a software designed to extract equilibrium constants from potentiometric and/or spectrophotometric titration data. HypSpec starts with an assumed complex formation scheme and uses a least-squares approach to derive the spectra of the complexes and the stability constants. χ^2^-test (chi-square) was applied, where the residuals follow a normal distribution (for a distribution approximately normal, the χ^2^-test value is around 12 or less). In all of the cases, χ^2^ ≤ 10 were found, as obtained by 3 independent measurement sets.

### Synthesis of Compound 4 (Johansson et al., 2003)

5.0 g (0.0300 mol) of compound **2** (Dalla Cort et al., 2006) and 5.97 g of potassium phthalimide **3** (0.0322 mol) were placed in a 250 mL round-bottom flask. The reagents were dissolved in 100 mL of DMF. The reaction mixture was allowed to stir at room temperature for 48 h and then was heated at 55°C for 4 h. The reaction mixture was cooled down to room temperature and diluted with 250 mL of EtOAc and washed with water (3 × 200 mL). The organic layer was dried over anhydrous Na_2_SO_4_, filtered and evaporated. The product was purified by silica gel column chromatography (CHCl_3_) to afford 3.37 g (40 % yield) of compound **4**. ^1^H NMR (500 MHz, CDCl_3_): δ = 10.99 (s, 1H); 9.88 (s, 1H); 7.86-7.84 (m, 2H); 7.73-7.71 (m, 2H); 7.67 (s, 1H); 7.65-7.63 (dd, *J* = 8.5 Hz, *J* = 2.0 Hz, 1H); 6.95 (d, *J* = 8.5 Hz, 1H); 4.82 (s, 2H).

### Synthesis of Compound 5

0.662 g (2.36 mmol) of compound **4** and 0.0171 g (0.036 mmol) of tetrabutylammonium tribromide were placed in a 20 mL round-bottom flask. In this flask 3.60 mL of 1,3-propanediol and 3.90 mL of triethyl orthoformate were added. The reaction mixture was allowed to stir at room temperature for 48 h, then diluted with 45 mL of EtOAc. The organic solution was washed with water (3 × 20 mL), dried over anhydrous Na_2_SO_4_, filtered and evaporated. The product was purified by silica gel column chromatography (cyclohexane/EtOAc 3:1) affording 0.732 g of white compound **5** (92% yield). ^1^H NMR (500 MHz, CDCl_3_): δ = 7.85 (s, 1H); 7.83–7.81 (m, 2H); 7.70–7.68 (m, 2H); 7.36–7.34 (dd, *J* = 8.5 Hz, *J* = 2.5 Hz, 1H); 7.28 (s, 1H); 6.84 (d, *J* = 8.5 Hz, 1H); 5.61 (s, 1H); 4.74 (s, 2H); 4.30–4.27 (m, 2H); 4.02–3.96 (m, 2H); 2.30– 2.20 (m, 1H); 1.52-1.48 (m, 1H). ^13^C (125 MHz, CDCl_3_): δ = 25.6, 31.4, 40.9, 67.2, 102.9, 117.4, 122.1, 123.2, 127.6, 128.6, 131.3, 132.1, 133.9, 154.9, 168.0.

### Synthesis of Compound 6

0.440 g (1.298 mmol) of compound **5** and 0.704 mL (0.713 mmol) of hydrazine monohydrate in 30 mL of EtOH were added in a round-bottomed flask of 50 mL. The reaction was heated at 90°C and monitored by TLC. After 20 min, the heating was interrupted. The reaction mixture was cooled down to room temperature and solvent was evaporated. The obtained white precipitate was diluted in 30 mL of water. The aqueous solution was extracted with CHCl_3_ (2 × 20 mL) which was previously passed through Al_2_O_3_ layer. The CHCl_3_ solution was dried over anhydrous Na_2_SO_4_, filtered and evaporated. Compound **6** (0.154 g, 57%) was obtained as a yellow oil. ^1^H NMR (500 MHz, CDCl_3_): δ = 7.18–7.16 (dd, *J* = 8.5 Hz, *J* = 2.0 Hz, 1H); 7.14 (s, 1H); 6.87–6.85 (d, *J* = 8.5 Hz, 1H); 5.65 (s, 1H); 4.33-4.30 (m, 2H); 4.04–3.99 (m, 2H); 3.77 (s, 2H); 2.32–2.30 (m, 1H); 1.54–1.50 (m, 1H). ^13^C NMR (125 MHz, CDCl_3_): 26.6; 45.7; 67.4; 102.4; 117.1; 122.2; 126.3; 129.2; 134.3; 154.0.

### Synthesis of Compound 8

0.394 g (0.598 mmol) of compound **7** (Gaware et al., 2017) were dissolved in 20 mL of anhydrous DMF, in a round-bottomed flask. To this stirred solution 0.273 g (0.718 mmol) of HATU was added, and the resulting mixture was left to stir under N_2_ atmosphere at room temperature for 20 min. Then, solution of 0.130 g (0.623 mmol) of compound **6** in 5 mL of anhydrous DMF was poured into the reaction mixture, which was stirred under N_2_ atmosphere and room temperature, for other 40 min. Finally, 0.105 mL (0.623 mmol) of *N*'*N*-diisopropylethylamine was added to the reaction mixture. The reaction was carried out in N_2_ atmosphere for 70 h. After this period, the reaction mixture was evaporated. The resulting precipitate was dissolved in 30 mL of CH_2_Cl_2_. The organic solution was washed with water (3 × 40 mL), dried over anhydrous Na_2_SO_4_ filtered and evaporated. Compound **8** was purified by silica gel column chromatography (CH_2_Cl_2_/MeOH 100:2) affording 80 mg (16% yield) of a purple solid. ^1^HNMR (500 MHz, CDCl_3_): δ = 8.85–8.61 (m, 8H); 8.29–8.26 (d, *J* = 8.0 Hz, 2H); 8.24–8.18 (m, 6H); 8.11–8.05 (d, *J* = 8.0 Hz, 2H); 7.90 (s, 1H); 7.78–7.72 (m, 9H); 7.37–7.33 (dd, *J* = 8.5 Hz, *J* = 2.0 Hz, 1H); 7.31 (d, *J* = 2.0 Hz, 1H); 6.96–6.93 (d, *J* = 8.5 Hz, 1H); 6.57 (s, 1H); 5.70 (s, 1H); 4.64 (d, 2H); 4.38–4.32 (m, 2H); 4.20–4.02 (m, 2H); 2.35–2.25 (m, 1H); 1.52-1.48 (m, 1H); −2.81 (s, 2H). ^13^C (125 MHz, CDCl_3_): δ = 25.7, 29.7, 43.9, 67.5, 102.9, 117.8, 118.5, 120.3, 125.3, 126.5 (x3), 126.7 (x3), 127.5 (x3) 127.7 (x3), 127.9, 129.2, 130.6, 131.7, 132.1, 134.4 (x2), 134.5, 134.6, 142.0, 142.7, 145.6, 150.3, 155.0.

### Synthesis of Compound 9

0.080 g (0.094 mmmol) of compound **8** were dissolved in 1.74 mL of TFA, in a round-bottomed flask, and the resulting mixture was allowed to stir at room temperature for 3 h. Then, 10 mL of diethyl ether were added to the organic solution affording a green precipitate, that was filtered and crystallized using MeOH, to give 50 mg (67% yield) of a red solid. ^1^HNMR (500 MHz, CDCl_3_): δ = 11.04 (s, 1H); 9.96 (s, 1H); 8.96–8.86 (m, 8H); 8.33–8.29 (d, *J* = 8.0 Hz, 2H); 8.24–8.19 (m, 6H); 8.26–8.22 (d, *J* = 8.0 Hz, 2H); 7.80–7.72 (m, 9H); 7.70 (d, *J* = 2.0 Hz, 1H); 7.68–7.64 (dd, *J* = 8.5 Hz, *J* = 2.0 Hz, 1H); 7.05 (d, *J* = 8.5 Hz, 1H); 6.77 (t, 1H); 4.76 (d, 2H); −2.77 (s, 2H). ^13^C (125 MHz, CDCl_3_): δ = 43.0, 118.0, 118.1, 120.4, 121.2, 125.0, 125.2, 126.5, 126.7, 127.5, 127.8, 128.5, 129.0, 131.4, 132.1, 132.2, 132.6, 132.8, 132.9, 134.4, 134.5, 134.6, 136.6, 136.7, 142.0, 142.8, 149.5, 150.1, 150.3, 150.4, 167.2, 196.3. MS (ESI): *m*/*z* = 790.2 [M + H]^+^.

### Synthesis of Salen-Bis-Porphyrin Ligand 10

In a round bottom flask, to a solution of 27.6 mg of salicylic-porphyrin **9** (0.0349 mmol) in absolute EtOH (10 mL) was added (1*R*,2*R*)-(+)-1,2-diphenylethylendiamine (3.7 mg, 0.0175 mmol). The reaction was stirred for 48 h at room temperature and monitored by TLC (CH_2_Cl_2_/CH_3_OH, 100:2). The reaction was quenched by evaporation of the solvent under reduced pressure, and salen-bis-porphyrin ligand **10** was purified by PLC (CH_2_Cl_2_/CH_3_OH, 100:2) to afford 24 mg of a red/purple solid compound (78% yield). ^1^HNMR (500 MHz, CDCl_3_): δ = 14.41 (bs, 2H); 8.86-8.74 (m, 16H); 8.30 (s, 2H); 8.25–8.07 (m, 20H); 7.77–7.66 (m, 20H); 7.29–7.15 (m, 12H); 6.97 (d, *J* = 8.5 Hz, 2H); 6.83 (t, 2H); 4.73 (s, 2H); 4.56 (d, 4H); −2.78 (s, 4H). ^13^C (125 MHz, CDCl_3_): δ = 43.3, 80.1, 117.2 (x2), 118.47, 118.53, 120.3 (x2), 120.5, 125.4 (x2), 126.3, 126.7 (x2), 127.7 (x3), 127.8 (x2), 128.4, 128.6, 131.0, 132.3, 133.5, 134.4 (x3), 134.5 (x2), 134.6, 139.0, 141.9, 142.0, 145.6, 160.4, 166.2, 167.4. MS (ESI): *m*/*z* = 1760.4 [M + H]^+^, *m*/*z* = 891.0 [M + Na]^2+^.

### Synthesis of Uranyl Salen-Bis-Porphyrin Complex 1

To a solution of **10** (22 mg, 0.0125 mmol) dissolved in absolute ethanol (10 mL) was added uranyl acetate (5.3 mg, 0.0125 mmol). The reaction was stirred overnight at room temperature, and the resulting solid was filtered and dried to yield 27 mg of uranyl salen-bis-porphyrin complex **1** as a red powder (98% yield). ^1^HNMR (500 MHz, DMSO-*d*_6_): δ = 9.49 (s, 2H); 9.33 (s, 2H) 8.82–8.76 (m, 16H); 8.32–8.20 (m, 20H); 7.83–7.80 (m, 20H); 7.74–7.59 (m, 8H); 7.21 (t, 2H); 7.15 (m, 2H); 7.05 (d, 2H); 6.35 (s, 2H); 4.67–4.57 (m, 4H); −2.93 (s, 4H). ^13^C (125 MHz, DMSO-*d*_6_): δ = 42.2, 79.5, 119.0 (x2), 120.1, 120.2 (x2), 120.3, 122.7, 125.9 (x3), 126.6 (x2), 127.0 (x3), 127.2 (x2), 127.4 (x3), 127.5, 128.1, 128.2, 133.7, 133.9, 134.2 (x3), 135.2, 141.1, 141.6, 144.0, 166.0, 168.6, 171.2. MS (ESI): *m*/*z* = 1058 [M+2EtOH+2H]^2+^; *m*/*z* = 1077 [M+2EtOH+Na+H]^2+^.

## Results and Discussion

**Scheme 1:**
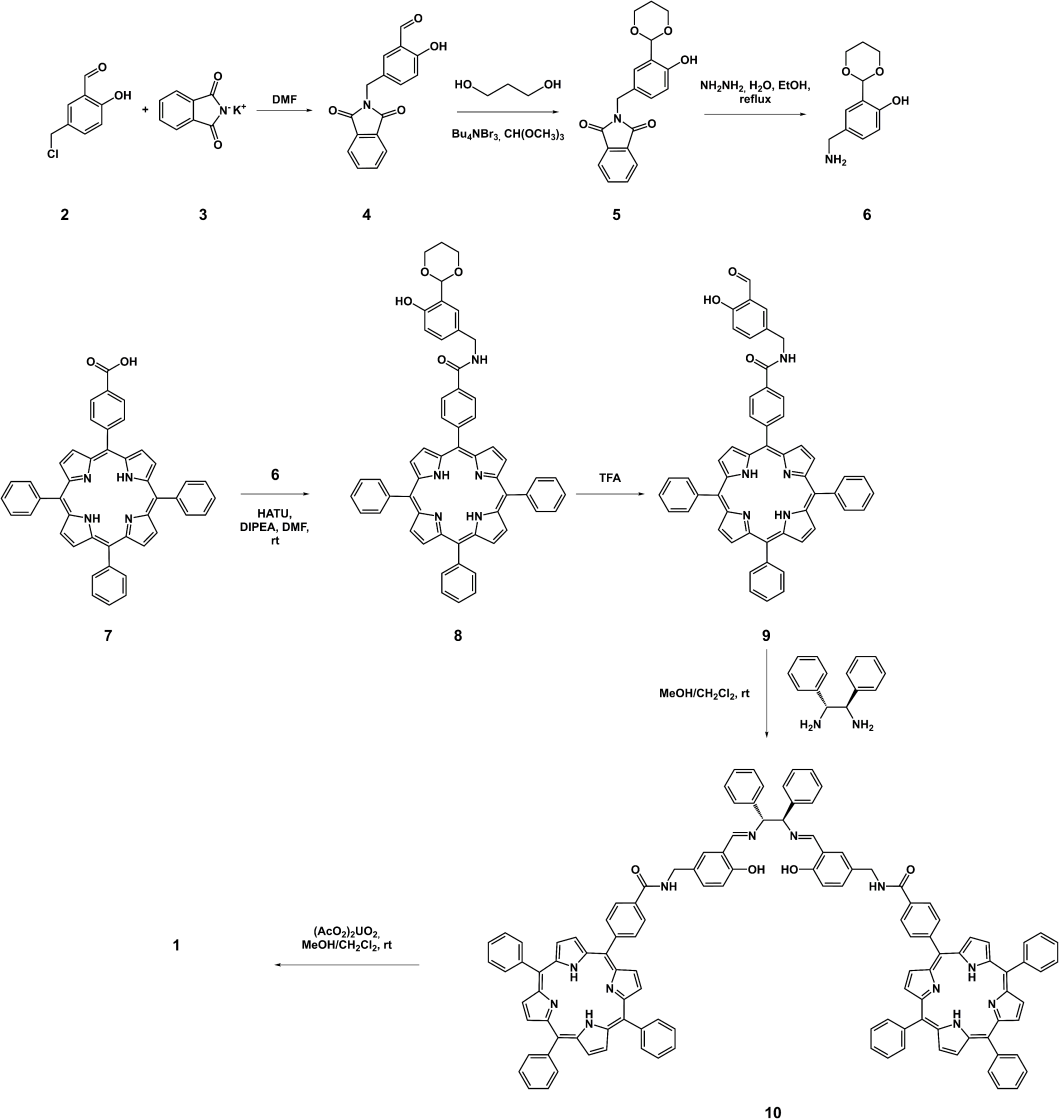
Synthesis of the uranyl salen-bis-porphyrin complex **1**.

Target uranyl salen-bis-porphyrin complex 1 was synthesized in seven steps starting from 5-Cl-methyl-salycilaldheyde 2 (Dalla Cort et al., 2006; Saffar-Telur, 2015) as shown in Scheme 1. In the first step potassium phtalimide was treated with compound 2 to yield compound 4 (40%), which was then reacted with 1,3-propandiol to afford the acetal intermediate 5 (92%). Conversion of the phthalimido moiety into an amino group by treatment with hydrazine, under standard Gabriel conditions, yielded the compound 6 (57%). The condensation reaction between compound 6 and 5-(4-Carboxyphenyl)-10,15,20-triphenylporphyrin 7 (Gaware et al., 2017) which was activated using HATU (Gangemi et al., 2015), afforded the porphyrin derivative 8 (16%), which was then treated with TFA to remove the acetal moiety and yield the salicylic-porphyrin 9 (67%). Condensation of 9 with the (1*R*,2*R*)-(+)-1,2-diphenylethylendiamine yielded salen ligand 10 (78%), which was finally converted into the corresponding salen complex 1 (98 %) by uranyl acetate. The proposed structures for this new chiral uranyl–salen complex and all the intermediates compounds are consistent with the ^1^H and ^13^C NMR spectroscopy data as well as the ESI mass spectrometry data (see Supplementary Material).

**Figure 2:**
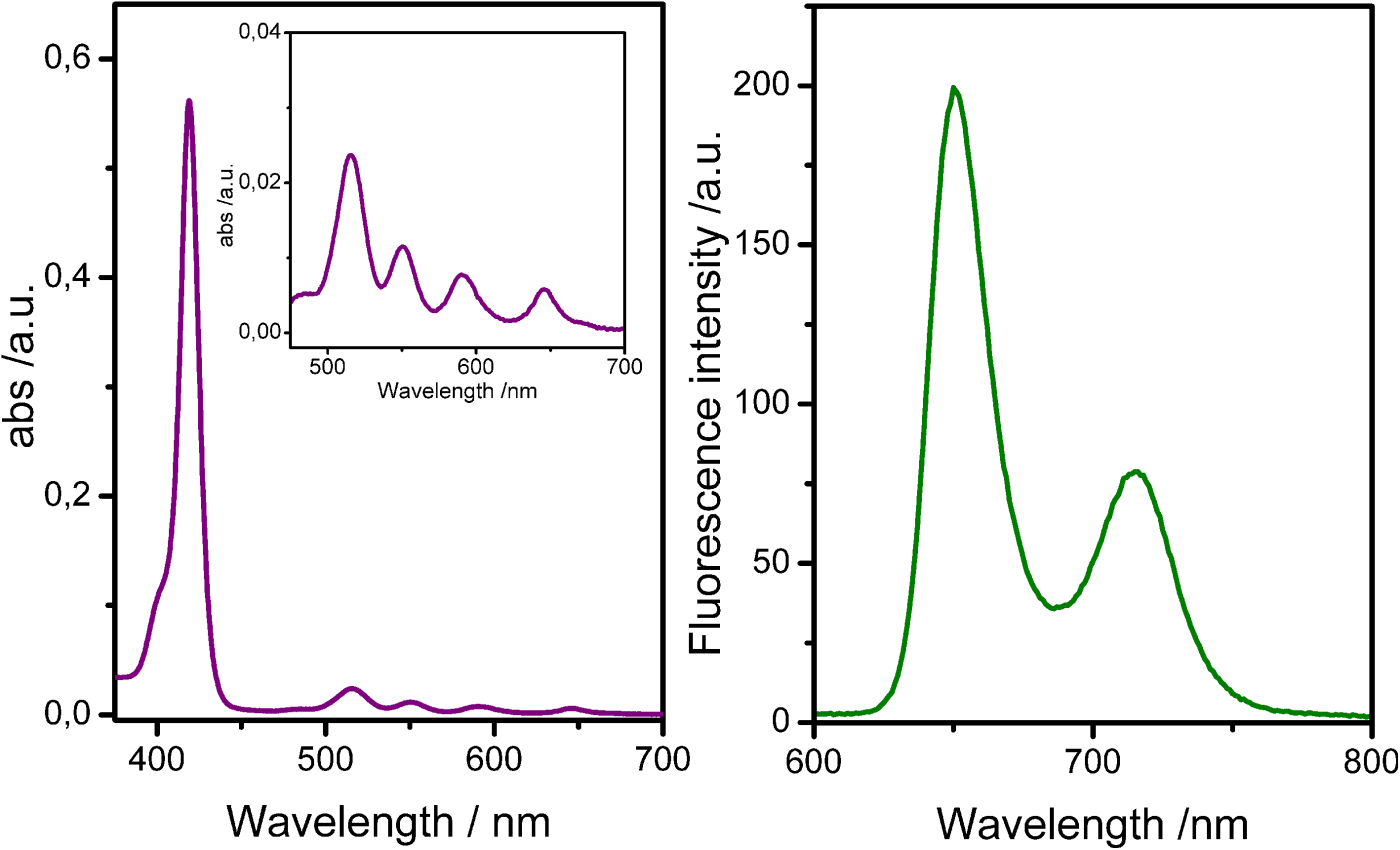
UV-Vis spectra **(Left)** and fluorescence spectra **(Right)** of uranyl salen-bis-porphyrin complex **1** in CHCl_3_ (1 μM).

The UV-vis spectrum of uranyl salen-bis-porphyrin complex **1** dissolved in CHCl_3_ solution shows an intense Soret band centered at 419 nm (ε = 6,26·10^5^ M^−1^ cm^−1^) and four Q-bands (515 nm; 550 nm; 590 nm; 646 nm) (Figure 2). The UV-vis spectrum is similar to the 5-(4-Methoxycarbonylphenyl)-10,15,20-triphenylporphyrin (TPPCOOMe, the precursor of 5-(4-Carboxyphenyl)-10,15,20-triphenylporphyrin **7**) in the same solvent (Rong et al., 2012), suggesting that the insertion of the salen-UO_2_ does not change the spectroscopic behavior of the porphyrin. Furthermore, there is no evidence of aggregation even at higher concentration (2.5 μM), as confirmed by UV-Vis and fluorescence data. In addition, the luminescence measurements are in accordance with literature data for similar porphyrins, with two main emission bands centered at 650 and 715 nm (λ_ex_ 350 nm), respectively (Figure 2).

**Figure 3:**
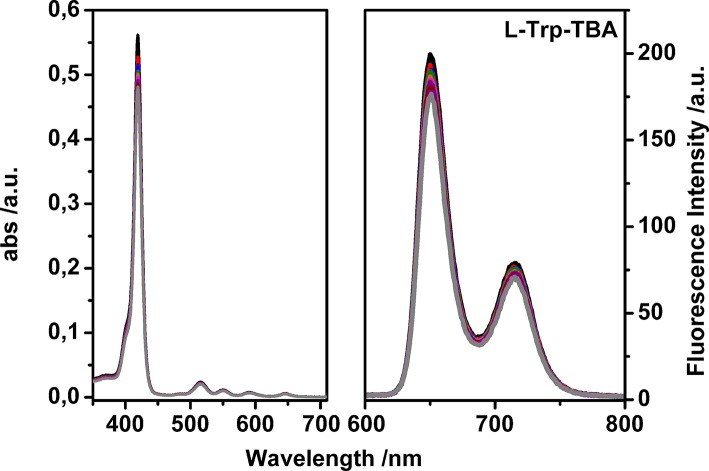
Representative UV-vis titration **(Left)** and fluorescence titration **(Right)** of uranyl salen-bis-porphyrin complex **1** with ***L*-Trp-TBA** (λ_ex_ = 350 nm in dry chloroform).

After proving the luminescent properties of uranyl salen-bis-porphyrin complex **1**, enantioselective recognition properties were evaluated by UV-vis and fluorescence measurements in chloroform, in particular for fluorescence titrations, following the emission changes at these two emission wavelengths of porphyrin moiety (650 nm and 715 nm, by using λ_ex_ 350 nm). Unfortunately, the fluorescence titrations showed a small intensity variation with all the selected amino acid guests except for the *L*-tryptophan derivative, which led to a poor data fitting. For that reasons, binding constant values between uranyl salen-bis-porphyrin complex **1** and amino acid derivatives were determined by UV-vis titrations, following a decrease of the absorption at 419 nm upon addition of increasing aliquots of guests. A representative example of UV-vis titration and the fluorescence titration of uranyl salen-bis-porphyrin complex **1** with L-Trp-TBA are shown in Figure 3.

Table 1 reports the pertinent binding constant values with selected amino acid derivatives, the detection limit observed (DL) and the corresponding enantiomeric excess. In all cases, binding constant values have been calculated using 1:1 stoichiometry, suggested by Job's plots (see Supplementary Material).

**Table 1:** Binding constant values *K* (M^−1^) with selected amino acid derivatives, detection limit observed (DL), and enantiomeric excess calculated by UV-vis titrations in dry chloroform at 25°C.

**Guest**	**DL^b^**	***K* (M^**−1**^)^a^**	**e.e**.
*D*-Phe-TBA	2.5 ppb	(8.13 ± 0.08) × 10^4^	*K_*L*_*/*K_*D*_* = 8.51
*L*-Phe-TBA	1.6 ppb	(6.92 ± 0.07) × 10^5^	
*D*-Ala-TBA	1.7 ppb	(9.77 ± 0.59) × 10^6^	*K_*D*_*/*K_*L*_* = 1.70
*L*-Ala-TBA	1.3 ppb	(5.75 ± 0.05) × 10^6^	
*D*-Trp-TBA	1.6 ppb	(4.33 ± 0.09) × 10^6^	*K_*D*_*/*K_*L*_* = 4.04
*L*-Trp-TBA	1.1 ppb	(1.07 ± 0.01) × 10^6^	

a *Calculated by HypSpec v1.1.33*.

b *Caculated by method of the calibration curve using the formula DL = 3σ/K, where σ is the standard deviation of the blank, and K is the slope of the calibration curve*.

Notably, due to the presence of the porphyrin moieties, receptor **1** is able to detect the amino acid guests at low concentration. In fact, 1 μM solution of uranyl salen-bis-porphyrin complex **1** is able to detect amino acid derivatives at very low concentrations (ppb).

Enantiomeric recognition is very efficient with the ***L*-Phe-TBA**, which is recognized more than 8 times respect to the *D*-enantiomer (*K*_*L*_/*K*_*D*_ = 8.51). A good enantioselectivity is also observed for the ***D*-** and ***L*-Trp-TBA** pair (*K*_*D*_/*K*_*L*_ = 4.04). Moreover, for the ***L*-Trp-TBA** we were able to determine the binding constant value by fluorescence titration (*K* (M^−1^ = 2.63 ± 0.03 × 10^6^), in good agreement with the value calculated by UV-vis titration. In particular, a decrease of the emission intensity has been observed, probably due to a photoinduced electron transfer mechanism (PET) (Trusso Sfrazzetto et al., 2016). Only the ***D*-** and ***L*-Ala-TBA** (*K*_*D*_/*K*_*L*_ = 1.70) pair shows a slight selective recognition. With the smaller amino acid guest, coordination to the uranyl metal center appears less susceptible to the different configurations of the carbon atom stereocenter and then the molecular recognition is less selective. With aromatic amino acid derivatives, the possibility for the carboxylate anion to bind the fifth equatorial coordination site of the uranyl metal and, at the same time, to exploit CH-π interactions with the salen moiety and the porphyrin macrocycles of receptor **1** might be responsible for the strong observed enantioselectivity. Moreover, the strong recognition for the ***L*-Phe-TBA** enantiomer is in contrast with respect to those observed with our previous receptors having the same configuration of the chiral diamine bridge (*R, R*) (Ballistreri et al., 2010; Amato et al., 2011; Pappalardo et al., 2012a; Forte et al., 2015). Probably, the presence of the two porphyrin arms not only increase the limit of detection by the presence of a fluorescence signal, but also plays a fundamental role in the recognition event.

## Conclusion

We have synthesized a new chiral uranyl salen complex bearing two porphyrin macrocycles and evaluated the enantiomeric recognition properties of this complex toward α-amino acid derivatives by UV-vis titrations. The presence of porphyrin arms lead to a receptor that possesses luminescence properties that are not quenched by the coordination with the uranyl cation, which decreases the fluorescence intensity in uranyl salen complexes. UV/Vis measurements and Job plots indicate the formation of 1:1 host–guest complexes. This receptor displays a very high selectivity toward amino acid derivatives, in particular for the two enantiomers of Phe-TBA. The two porphyrin macrocycles play a key role in the enantioselectivity interacting through CH–π with the aromatic moiety of aminoacids, leading to high binding affinities. Work is in progress in our laboratory to better understand the rules governing the interactions of this salen receptor with amino acid guests in order to design new host–guest systems that possess luminescence properties.

## Data Availability Statement

All datasets generated for this study are included in the article/Supplementary Material.

## Author Contributions

CG, UR, FC, and AP performed the synthesis of uranyl porphyrin-salen complex. GT acquired UV-vis and luminescence data. AP writing-original draft preparation. CG, UR, FC, GT, and AP writing-review and editing.

### Conflict of Interest

The authors declare that the research was conducted in the absence of any commercial or financial relationships that could be construed as a potential conflict of interest. The reviewer SG declared a past co-authorship with one of the authors AP to the handling editor.

## References

[B1] Amato M. E. Ballistreri F. P. D'Agata S. Pappalardo A. Tomaselli G. A. Toscano R. M. (2011). Enantioselective molecular recognition of chiral organic ammonium ions and amino acids using cavitand–salen-based receptors. Eur. J. Org. Chem. 21, 5674–5680. 10.1002/ejoc.201100955

[B2] Amato M. E. Ballistreri F. P. Gentile S. Pappalardo A. Tomaselli G. A. Toscano R. M. (2010). Recognition of achiral and chiral ammonium salts by neutral ditopic receptors based on chiral salen-UO_2_ macrocycles. J. Org. Chem. 75, 1437–1443. 10.1021/jo902328y 20143850

[B3] Amato M. E. Ballistreri F. P. Pappalardo A. Sciotto D. Tomaselli G. A. Toscano R. M. (2007). Synthesis and conformational aspects of 20- and 40-membered macrocyclic mono and dinuclear uranyl complexes incorporating salen and (R)-BINOL units. Tetrahedron 63, 9751–9757. 10.1016/j.tet.2007.07.014

[B4] Baleizao C. Garcia H. (2006). Chiral salen complexes: an overview to recoverable and reusable homogeneous and heterogeneous catalysts. Chem. Rev. 106, 3987–4043. 10.1021/cr050973n 16967927

[B5] Ballistreri F. P. Gangemi C. M. Pappalardo A. Tomaselli G. A. Toscano R. M. Trusso Sfrazzetto G. (2016). (Salen)Mn(III) catalyzed asymmetric epoxidation reactions by hydrogen peroxide in water: a green protocol. Int. J. Mol. Sci. 17:1112. 10.3390/ijms17071112 27420047 PMC4964487

[B6] Ballistreri F. P. Lombardo G. Pappalardo A. Punzo F. Thompson A. Tomaselli G. A.. (2012). An integrated X-ray and molecular dynamics study of uranyl–salen structures and properties. Dalton Trans. 41, 1951–1960. 10.1039/C1DT11758K 22183509

[B7] Ballistreri F. P. Pappalardo A. Toscano R. M. Tomaselli G. A. Trusso Sfrazzetto G. (2010). Heteroditopic chiral uranyl–salen receptor for molecular recognition of amino acid ammonium salts. Eur. J. Org. Chem. 20, 3806–3810. 10.1002/ejoc.201000566

[B8] Ballistreri F. P. Toscano R. M. Amato M. E. Pappalardo A. Gangemi C. M. A. Spidalieri S. (2018). A new Mn–salen micellar nanoreactor for enantioselective epoxidation of alkenes in water. Catalysts 8:129 10.3390/catal8040129

[B9] Beletskaya I. Tyurin V. S. Tsivadze A. Y. Guilard R. Stern C. (2009). Supramolecular chemistry of metalloporphyrins. Chem. Rev. 109, 1659–1713. 10.1021/cr800247a 19301872

[B10] Brancatelli G. Geremia S. Notti A. Pappalardo A. Trusso Sfrazzetto G. (2013). Mono- and dinuclear uranyl(VI) complexes with chiral Schiff base ligand. Inorg. Chim. Acta 396, 25–29. 10.1016/j.ica.2012.12.034

[B11] Cozzi P. G. (2004). Metal–Salen Schiff base complexes in catalysis: practical aspects. Chem. Soc. Rev. 33, 410–421. 10.1039/B307853C 15354222

[B12] Dalla Cort A. Mandolini L. Pasquini C. Schiaffino L. (2006). A novel ditopic zinc-salophen macrocycle: a potential two-stationed wheel for [2]-pseudorotaxane. Org. Biomol. Chem. 4, 4543–4546. 10.1039/b613705a 17268651

[B13] Drain C. M. Varotto A. Radiovojevic I. (2009). Self-organized porphyrinic materials. Chem. Rev. 109, 1630–1658. 10.1021/cr8002483 19253946 PMC2681784

[B14] D'Urso A. Tudisco C. Ballistreri F. P. Condorelli G. G. Randazzo R. Tomaselli G. A.. (2014). Enantioselective extraction mediated by a chiral cavitand–salen covalently assembled on a porous silicon surface. Chem. Commun. 50, 4993–4496. 10.1039/C4CC00034J 24504122

[B15] Forte G. D'Urso A. Ballistreri F. P. Toscano R. M. Tomaselli G. A. Trusso Sfrazzetto G. (2015). Enantiomeric recognition of α-amino acid derivatives by chiral uranyl-salen receptors. Tetrahedron Lett. 56, 2922–2926. 10.1016/j.tetlet.2015.04.092

[B16] Gangemi C. M. A. Randazzo R. Fragal,à M. E. Tomaselli G. A. Ballistreri F. P. Pappalardo A. (2015). Hierarchically controlled protonation/aggregation of a porphyrin-spermine derivative. New J. Chem. 39, 6722–6725. 10.1039/C5NJ01264C

[B17] Gaware V. S. Hakerud M. Juzeniene A. Høgset A. Berg K. Másson M. (2017). Endosome targeting meso-tetraphenylchlorin–chitosan nanoconjugates for photochemical internalization. Biomacromolecules 18, 1108–1126. 10.1021/acs.biomac.6b01670 28245649

[B18] Jacobsen E. N. (1993). “Asymmetric catalytic epoxidation of unfunctionalized olefins,” in Catalytic Asymmetric Synthesis, ed Ojima I. (New York, NY: VCH, 159–202.

[B19] Jacobsen E. N. (2000). Asymmetric catalysis of epoxide ring-opening reactions. Acc. Chem. Res. 33, 421–431. 10.1021/ar960061v 10891060

[B20] Johansson A. Abrahamsson M. Magnuson A. Huang P. Mårtensson J. Styring S.. (2003). Synthesis and photophysics of one mononuclear Mn(III) and one dinuclear Mn(III,III) complex covalently linked to a ruthenium(II) tris(bipyridyl) complex. Inorg. Chem. 42, 7502–7511. 10.1021/ic0344822 14606845

[B21] Katsuki T. (1995). Catalytic asymmetric oxidations using optically active (salen)manganese(III) complexes as catalysts. Coord. Chem. Rev. 140, 189–214. 10.1016/0010-8545(94)01124-T

[B22] Katsuki T. (2000). “Asymmetric oxidations and related reactions: asymmetric epoxidation of unfunctionalized olefins and related reactions,” in Catalytic Asymmetric Synthesis, 2nd Edn, ed Ojima I. (New York, NY: Wiley-VCH, 287–325.

[B23] La Paglia Fragola V. Lupo F. Pappalardo A. Trusso Sfrazzetto G. Toscano R. M. Ballistreri F. P. (2012). A surface-confined O = MnV(salen) oxene catalyst and high turnover values in asymmetric epoxidation of unfunctionalized olefins. J. Mater. Chem. 22, 20561–20561. 10.1039/c2jm34847k

[B24] McGarrigle E. M. Gilheany D. G. (2005). Chromium- and manganese-salen promoted epoxidation of alkenes. Chem. Rev. 105, 1563–1602. 10.1021/cr0306945 15884784

[B25] Pappalardo A. Amato M. E. Ballistreri F. P. Tomaselli G. A. Toscano R. M. Trusso Sfrazzetto G. (2012a). Pair of diastereomeric uranyl salen cavitands displaying opposite enantiodiscrimination of α-amino acid ammonium salts. J. Org. Chem. 77, 7684–7687. 10.1021/jo301098d 22892015

[B26] Pappalardo A. Ballistreri F. P. Li Destri G. Mineo P. G. Tomaselli G. A. Toscano R. M. (2012b). Supramolecular polymer networks based on calix[5]arene tethered poly(*p*-phenyleneethynylene). Macromolecules 45, 7549–7556. 10.1021/ma3015239

[B27] Puglisi R. Ballistreri F. P. Gangemi C. M. A. Toscano R. M. Tomaselli G. A. Pappalardo A. (2017). Chiral Zn–salen complexes: a new class of fluorescent receptors for enantiodiscrimination of chiral amines. New J. Chem. 41, 911–915. 10.1039/C6NJ03592B

[B28] Puglisi R. Mineo P. G. Pappalardo A. Gulino A. Trusso Sfrazzetto G. (2019). Supramolecular detection of a nerve agent simulant by fluorescent Zn–salen oligomer receptors. Molecules 24:2160. 10.3390/molecules24112160 31181723 PMC6600340

[B29] Puglisi R. Pappalardo A. Gulino A. Trusso Sfrazzetto G. (2018). Supramolecular recognition of CWAs simulant by metal-salen complexes: the first multi-topic approach. Chem. Commun. 54, 11156–11159. 10.1039/C8CC06425C 30226513

[B30] Rong Y. Chen P. Wang D. Liu M. (2012). Porphyrin assemblies through the air/water interface: effect of hydrogen bond, thermal annealing, and amplification of supramolecular chirality. Langmuir 28, 6356–6363. 10.1021/la3005294 22444117

[B31] Saffar-Telur A. (2015). Direct covalent attachment of Mn(III) salophen complex to the hydroxyapatite-encapsulated γ- Fe_2_O_3_ nanocrystallites: an efficient magnetic and reusable catalyst for oxidation of alcohols. RSC Adv. 5, 70577–70585. 10.1039/C5RA08594B

[B32] Trusso Sfrazzetto G. Millesi S. Pappalardo A. Toscano R. M. Ballistreri F. P. Tomaselli G. A. (2015). Olefin epoxidation by a (salen)Mn(III) catalyst covalently grafted on glass beads. Cat. Sci. Techn. 5, 673–679. 10.1039/C4CY00831F

[B33] Trusso Sfrazzetto G.;, Satriano, C.;, Tomaselli, G. A.;, and Rizzarelli, E. (2016). Synthetic fluorescent probes to map metallostasis and intracellular fate of zinc and copper. Coord. Chem. Rev. 311, 125–167. 10.1016/j.ccr.2015.11.012

[B34] Wezenberg S. J. Kleij A. W. (2008). Material applications for salen frameworks. Angew. Chem., Int. Ed. 47, 2354–2364. 10.1002/anie.200702468 18306204

[B35] Whiteoak C. J. Salassa G. Kleij A. W. (2012). Recent advances with π-conjugated salen systems. Chem. Soc. Rev. 41, 622–631. 10.1039/C1CS15170C 21842040

[B36] Yoon T. P. Jacobsen E. N. (2003). Privileged chiral catalysts. Science 299, 1691–1693. 10.1126/science.1083622 12637734

[B37] Zammataro A. Gangemi C. M. A. Pappalardo A. Toscano R. M. Puglisi R. Nicotra G.. (2019). Covalently functionalized carbon nanoparticles with a chiral Mn-Salen: a new nanocatalyst for enantioselective epoxidation of alkenes. Chem. Commun. 55, 5255–5258. 10.1039/C9CC01825E 30990489

