# Supplementary material for: Enantiomeric Recognition of α-Aminoacids by a Uranyl Salen-Bis-Porphyrin Complex
Source: Front Chem. 2019 Dec 3;7:836. doi: 10.3389/fchem.2019.00836 (PMC6902086; doi:10.3389/fchem.2019.00836)
Supplement: Supplementary file 1 [file Data_Sheet_1.PDF]

Supplementary Material

1 Supplementary Data

Determination of Stoichiometry. Stoichiometry of the supramolecular complexes were investigated by the Job's plot method, using spectrophotometric measurements. The samples were prepared by mixing equimolecular stock solutions (1.0×10^{-3} M) of the appropriate host and guest to cover the whole range of molar fractions, keeping constant the total concentration (1×10^{-5} M). The changes in absorbance compared to uncomplexed receptor species ($\Delta A \times \chi^{-1}$) were calculated and reported versus the receptor mole fraction (χ). These plots show invariably a maximum at 0.5 mol fraction of receptor, thus suggesting its 1:1 complex formation.

UV- vis titration with D-Phe-TBA

```
Converged in 4 iterations with sigma = 3,6058E-03

                          standard
Log beta     value        deviation
AB           4.9057       0.051
```

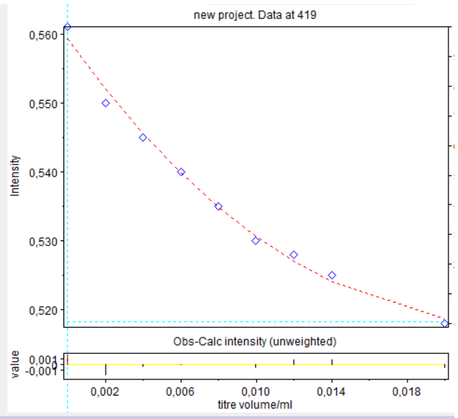

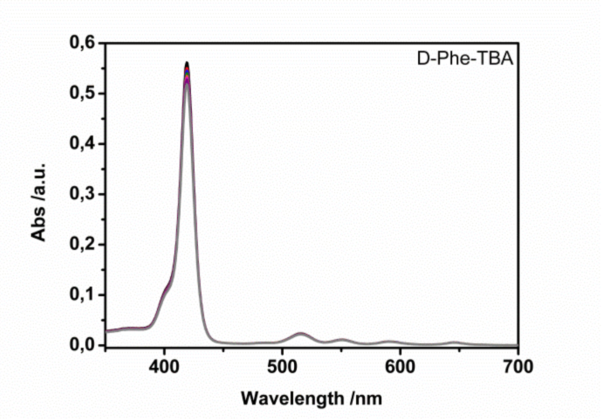

UV- vis titration with L-Phe-TBA

```
Converged in 4 iterations with sigma = 8,2041E-04
```

Log beta	value	standard deviation
AB	5.843	0.0173

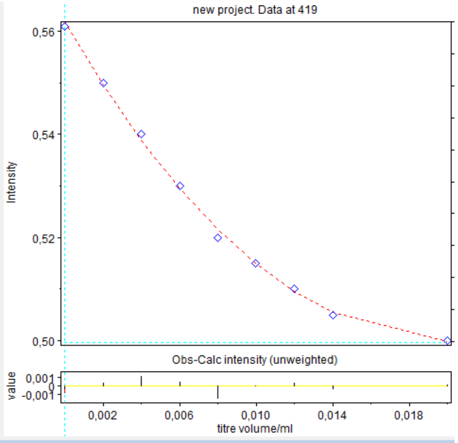

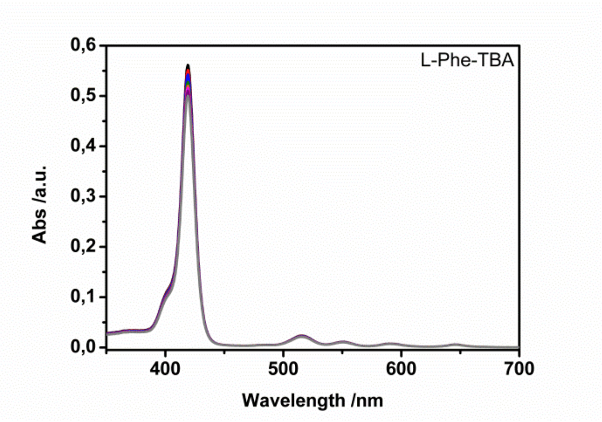

UV- vis titration with D-Ala-TBA

```
Converged in 5 iterations with sigma = 3,9894E-04
```

Log beta	value	standard deviation
AB	6.9923	0.0423

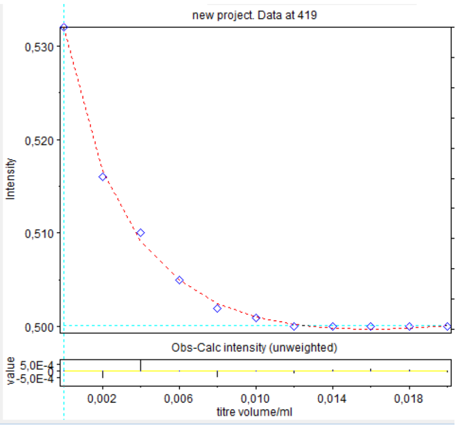

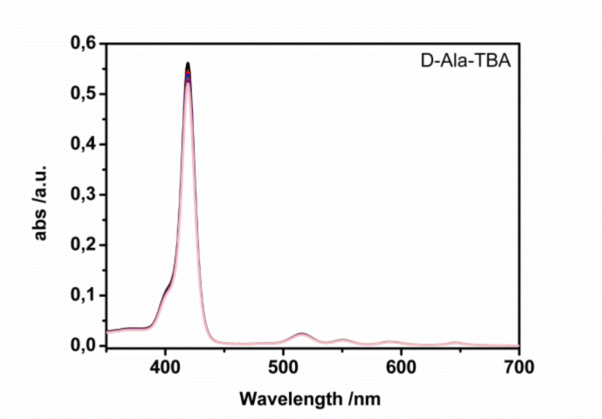

UV- vis titration with L-Ala-TBA

Converged in 4 iterations with sigma = 4,4236E-04

Log beta	value	standard deviation
AB	6.7619	0.028

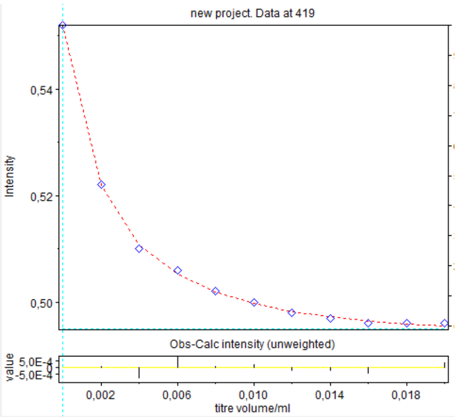

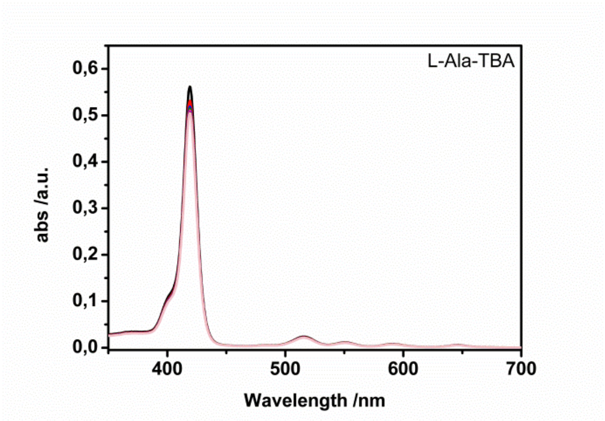

UV- vis titration with D-Trp-TBA

Converged in 1 iterations with sigma = 6,5916E-04

Log beta	value	standard deviation
AB	6.6312	0.034

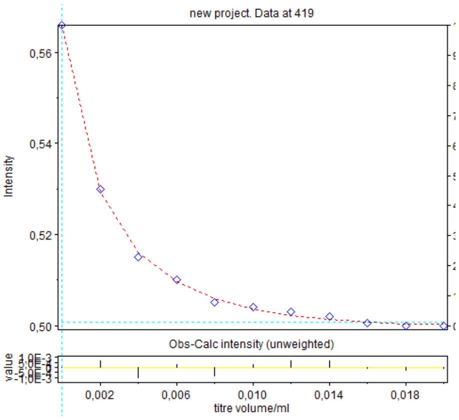

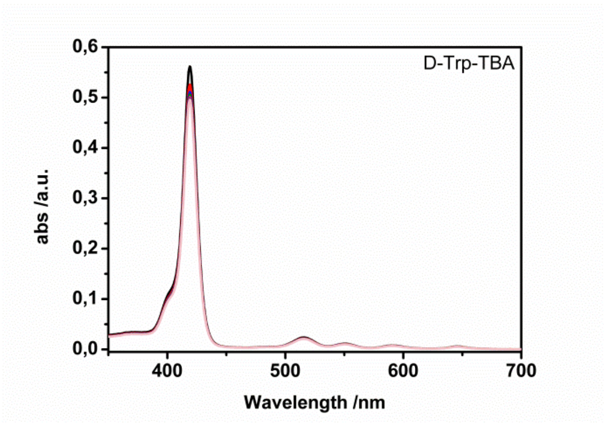

UV- vis titration with L-Trp-TBA

```
Converged in 4 iterations with sigma = 1,1005E-03
```

Log beta	value	standard deviation
AB	6.0267	0.0649

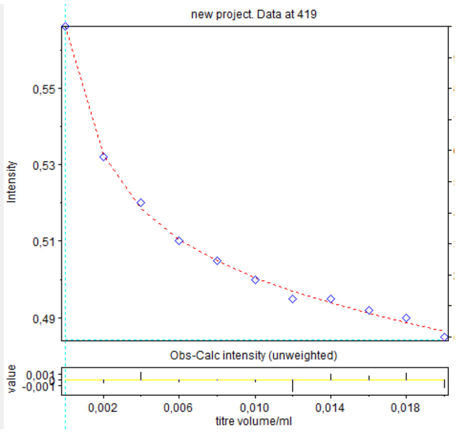

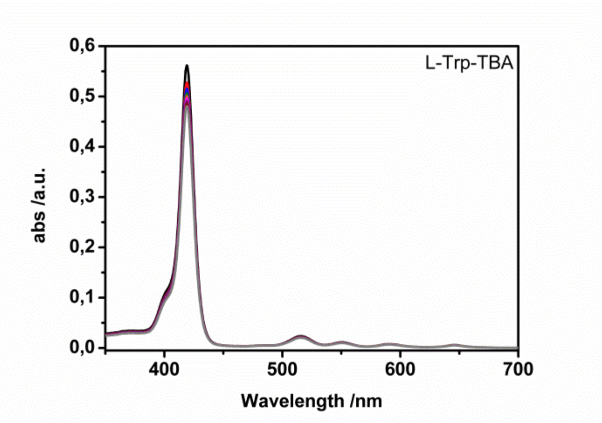

Fluorescence titration with L-Trp-TBA

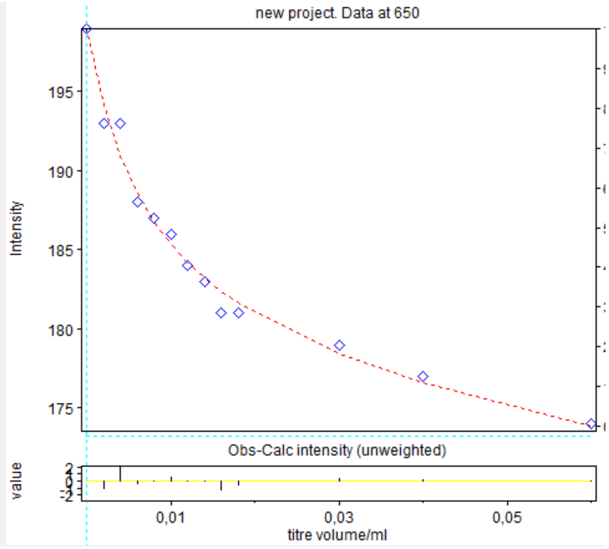

HypSpec output file

Converged in 1 iterations with sigma = 0,87734

Log beta	value	standard deviation
AB	6.4151	0.0703

Job's Plot with D-Phe-TBA

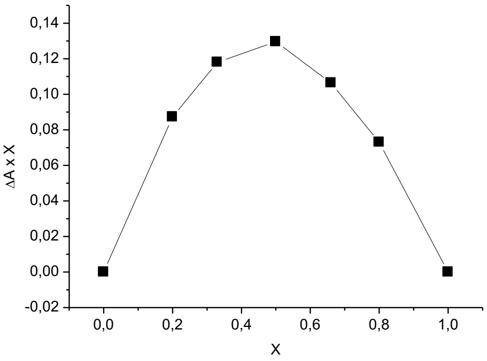

Job's Plot with D-Ala-TBA

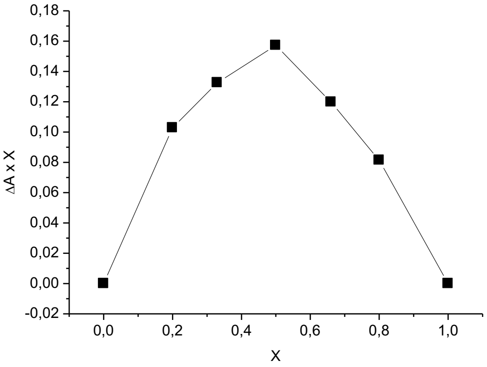

Job's Plot with D-Trp-TBA

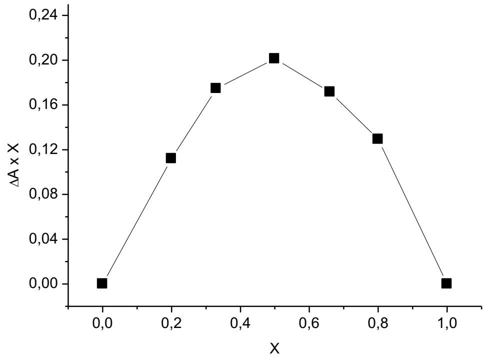

2 Supplementary Figures and Tables

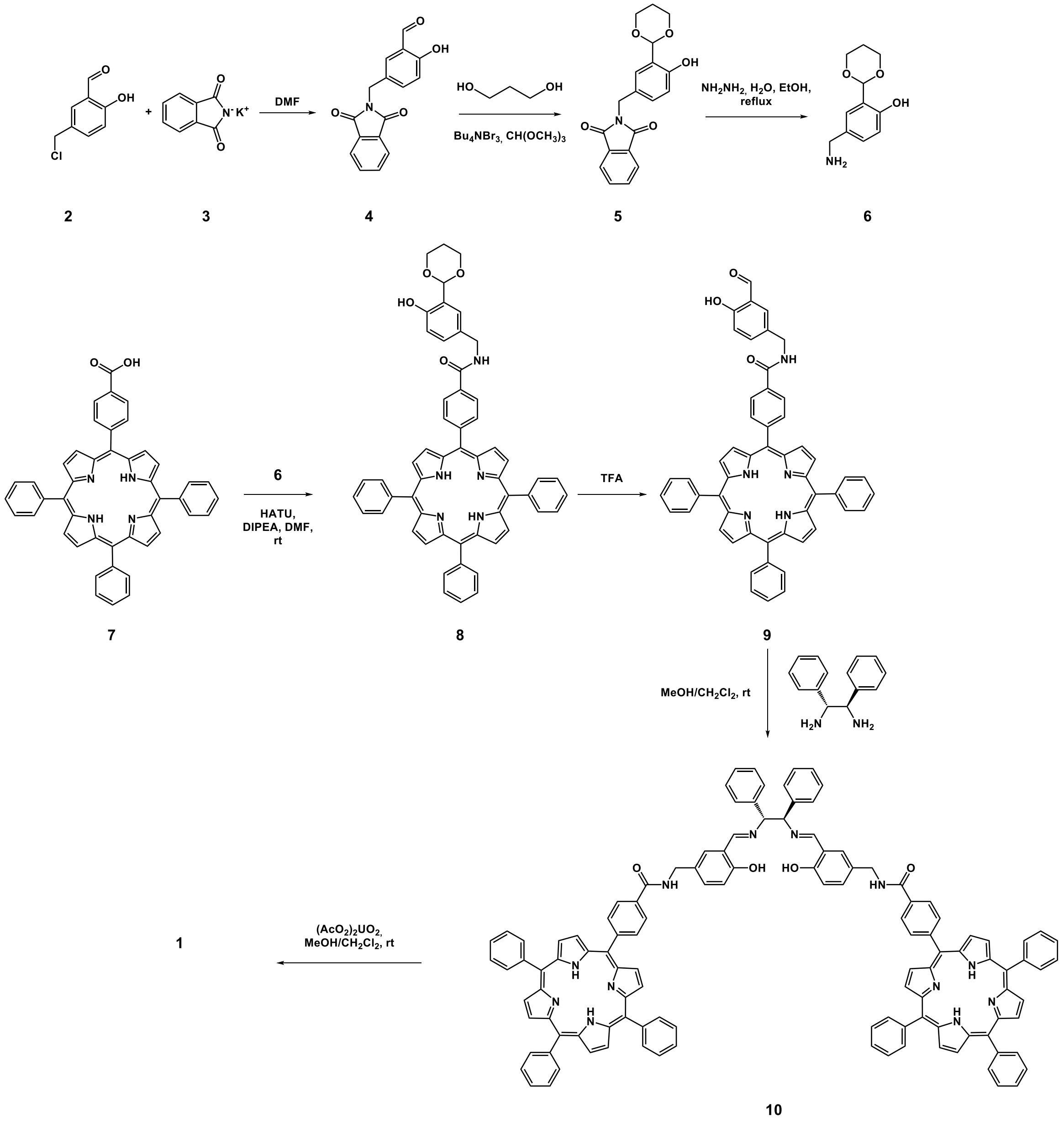

Supplementary Figure 1. Synthesis of the uranyl salen-bis-porphyrin complex **1**

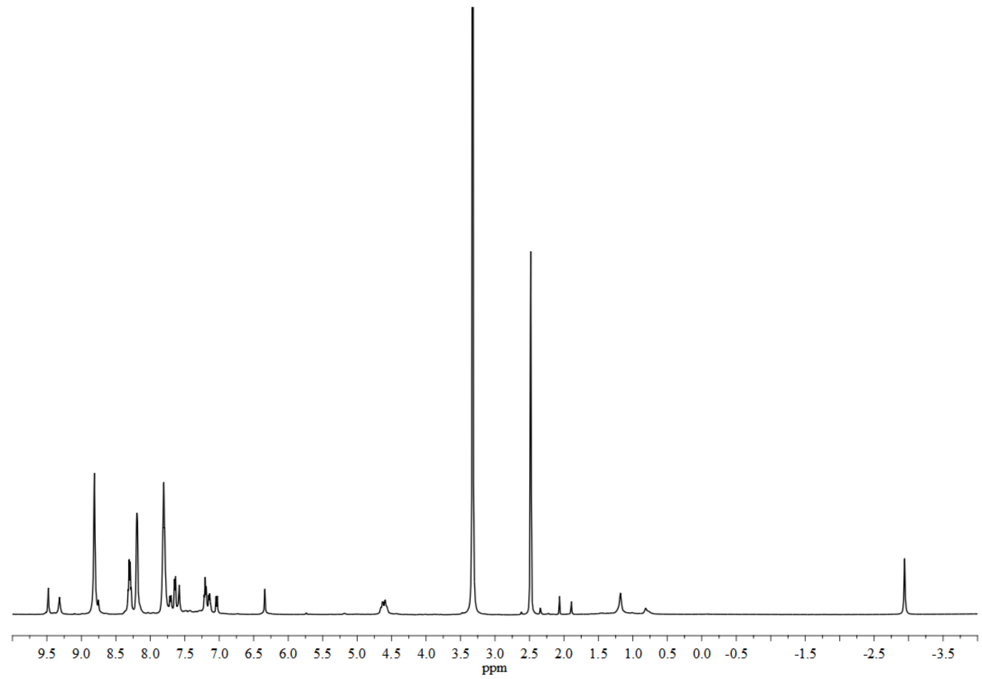

Supplementary Figure 2. ^{1}H-NMR of receptor **1** in DMSO-d_6

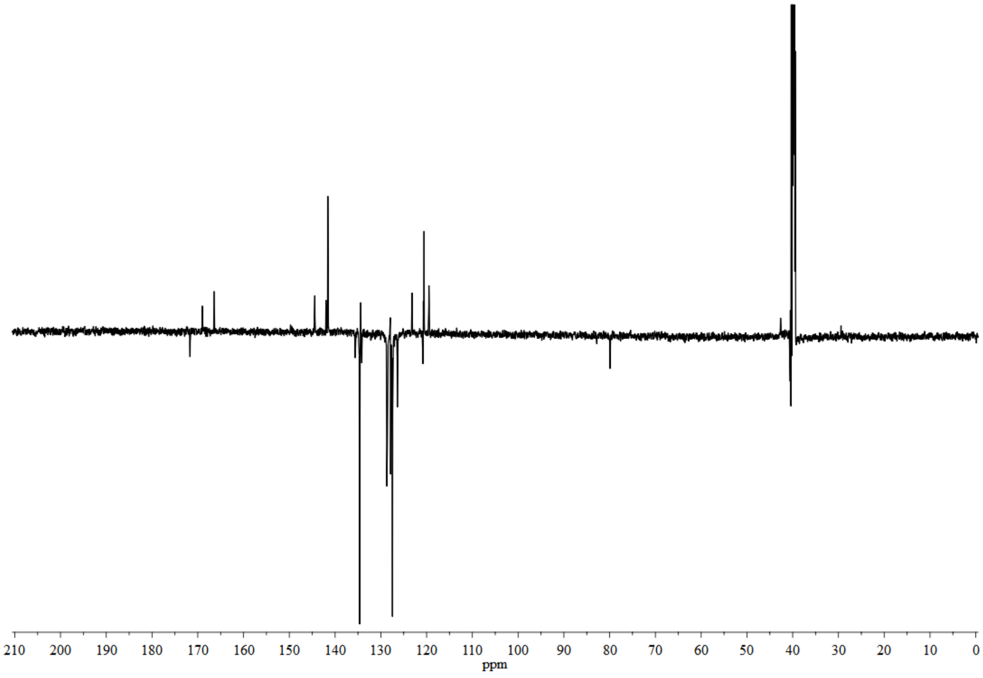

Supplementary Figure 3. APT of receptor **1** in DMSO-d_6

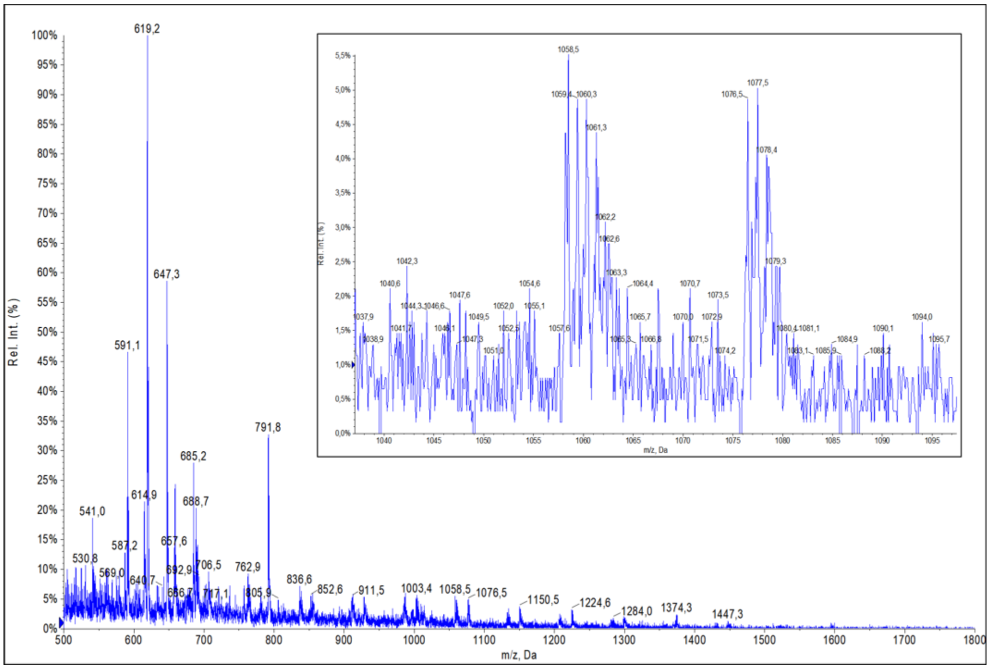

Supplementary Figure 4 ESI-MS of receptor **1**

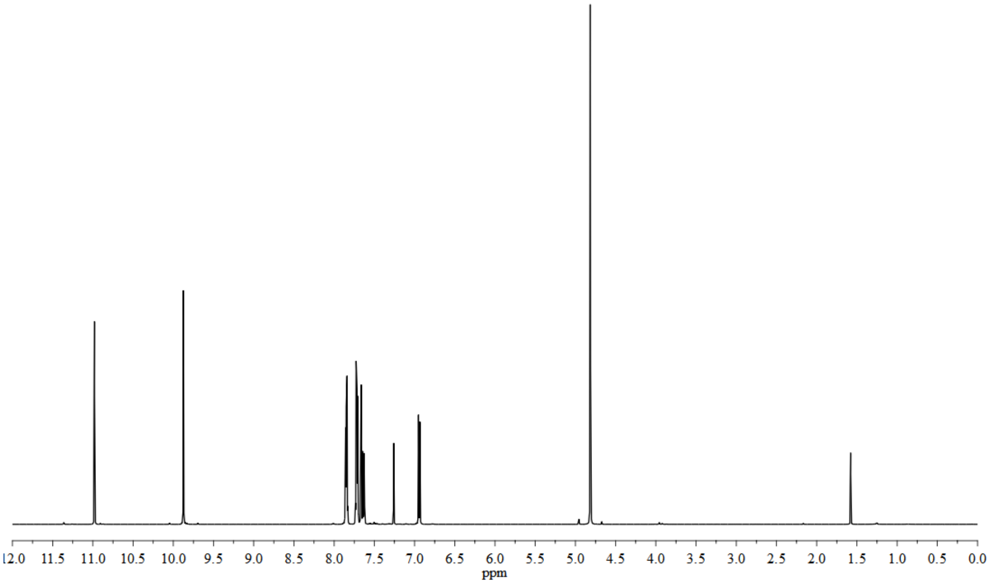

Supplementary Figure 5. ^{1}H-NMR of compound **4** in $CDCl_3$

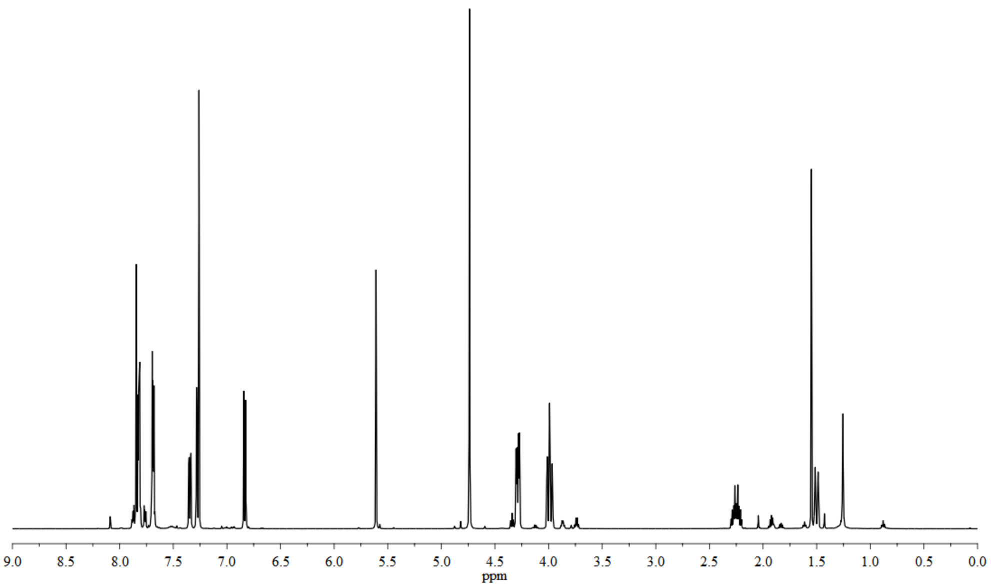

Supplementary Figure 6. ^{1}H-NMR of compound **5** in $CDCl_3$

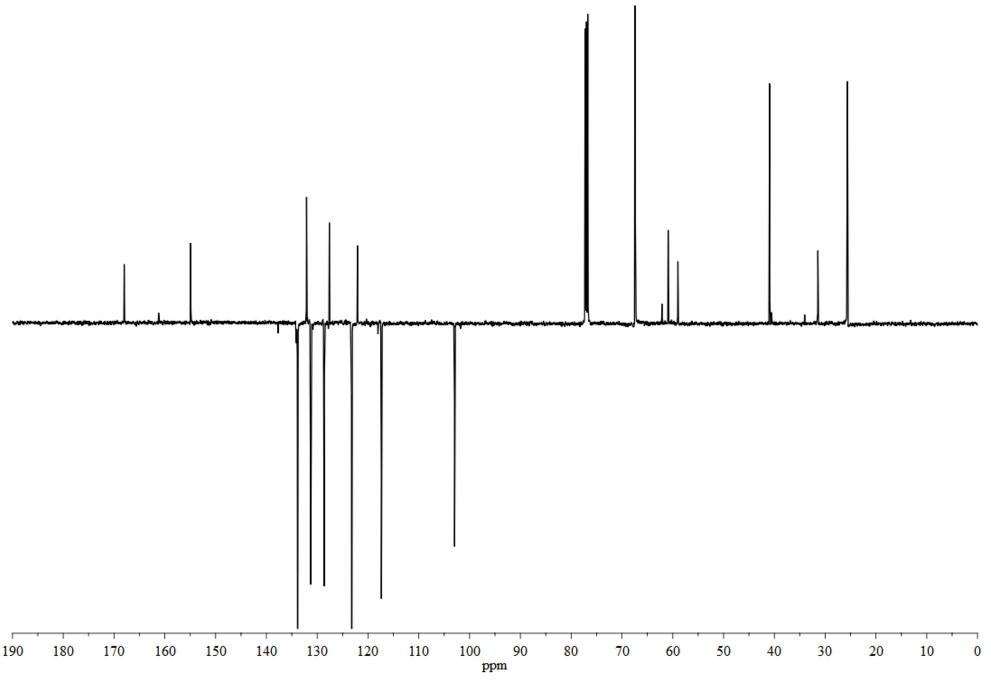

Supplementary Figure 7. APT of compound **5** in $CDCl_3$

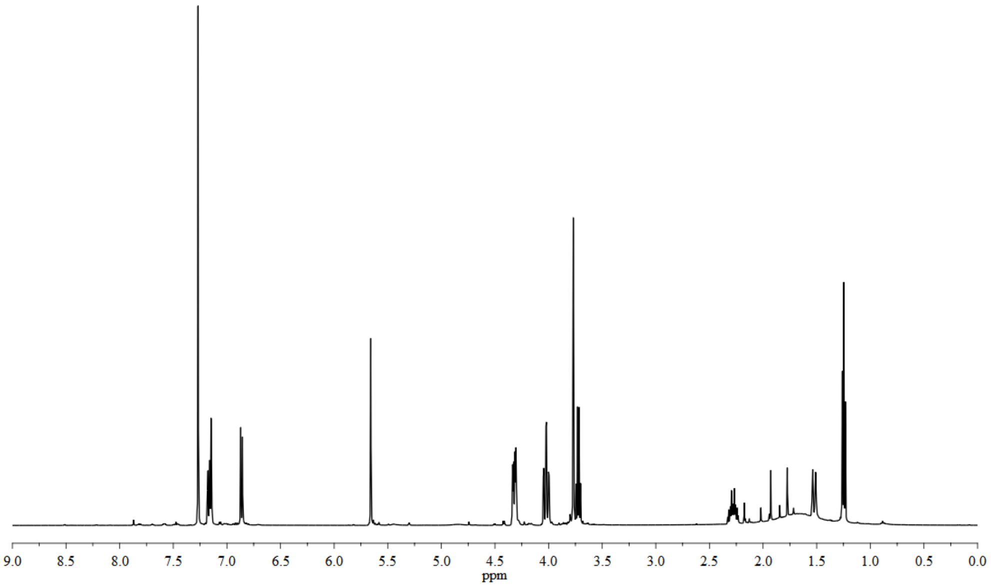

Supplementary Figure 8. ^{1}H-NMR of compound **6** in $CDCl_3$

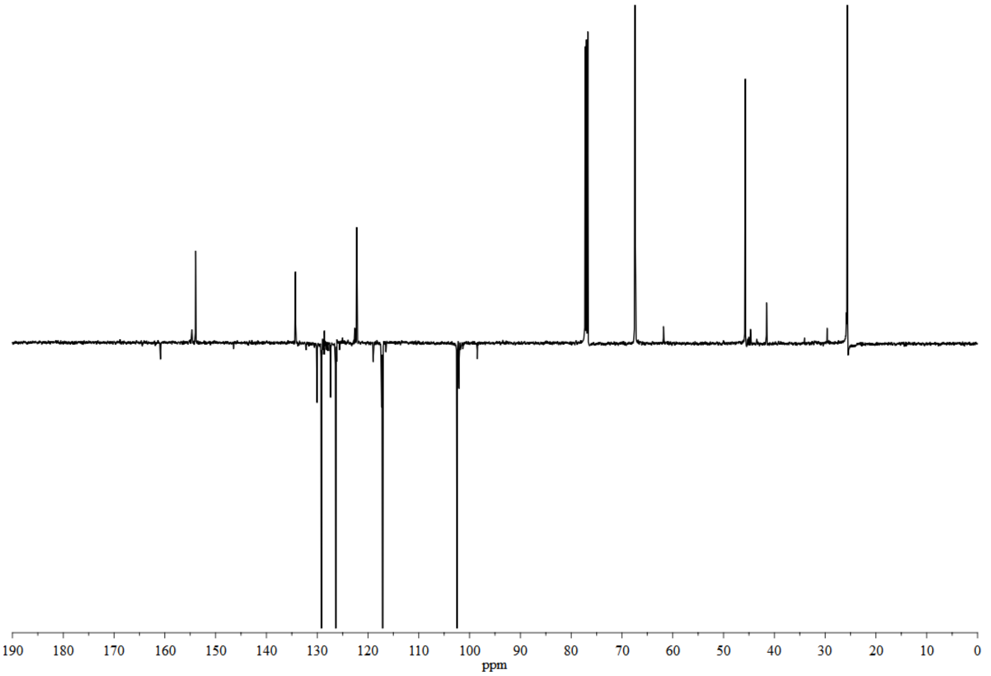

Supplementary Figure 9. APT of compound **6** in $CDCl_3$

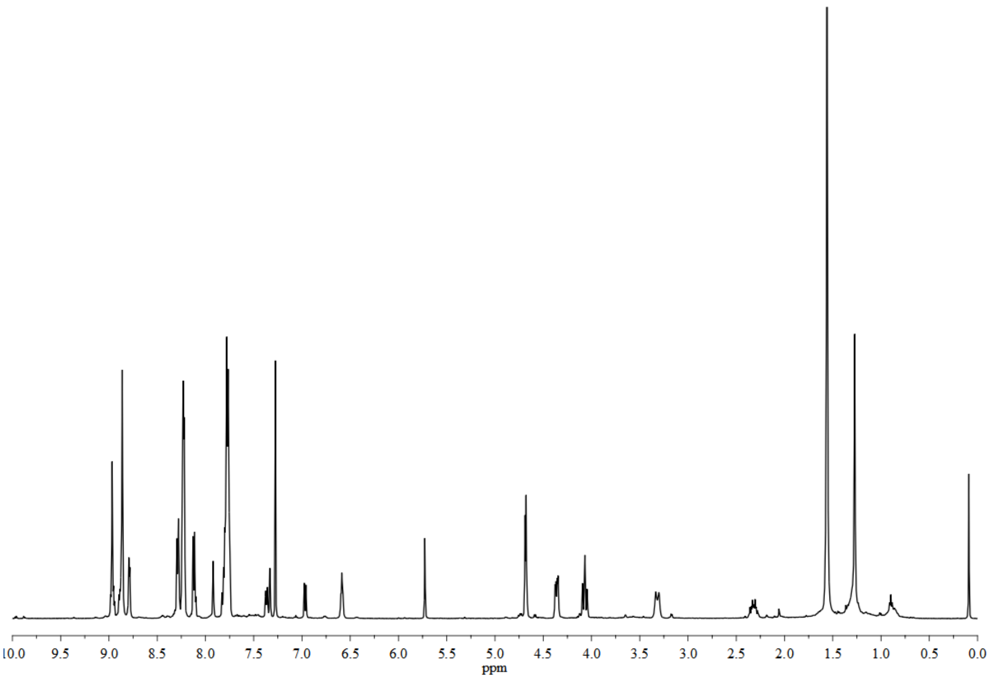

Supplementary Figure 10. ^{1}H-NMR of compound **8** in $CDCl_3$

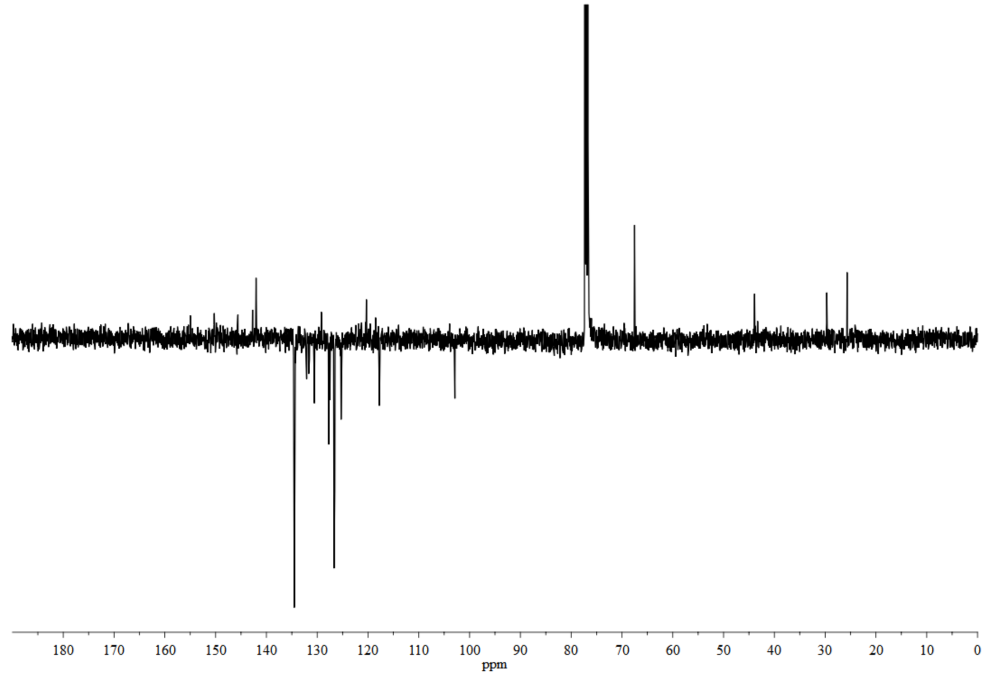

Supplementary Figure 11. APT of compound **8** in $CDCl_3$

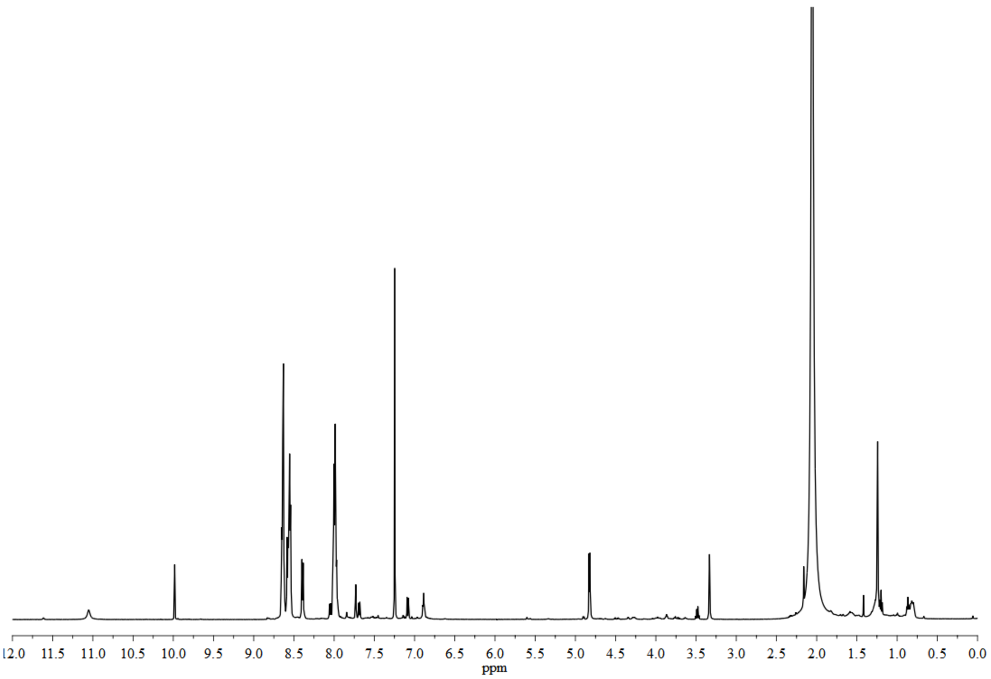

Supplementary Figure 12. ^{1}H-NMR of compound **9** in $CDCl_3$

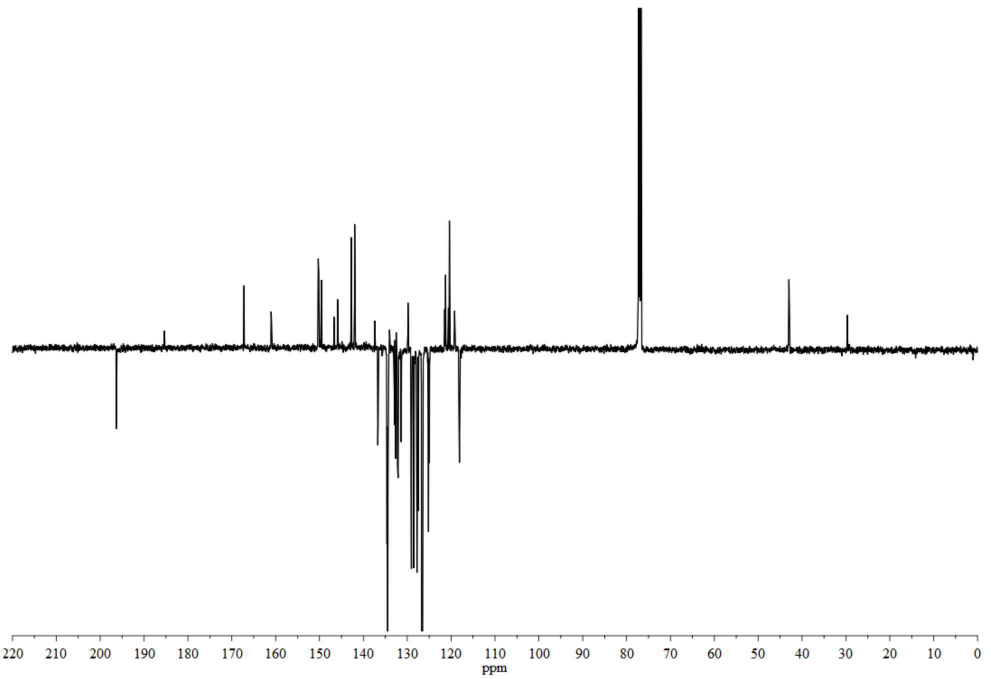

Supplementary Figure 13. APT of compound **9** in $CDCl_3$

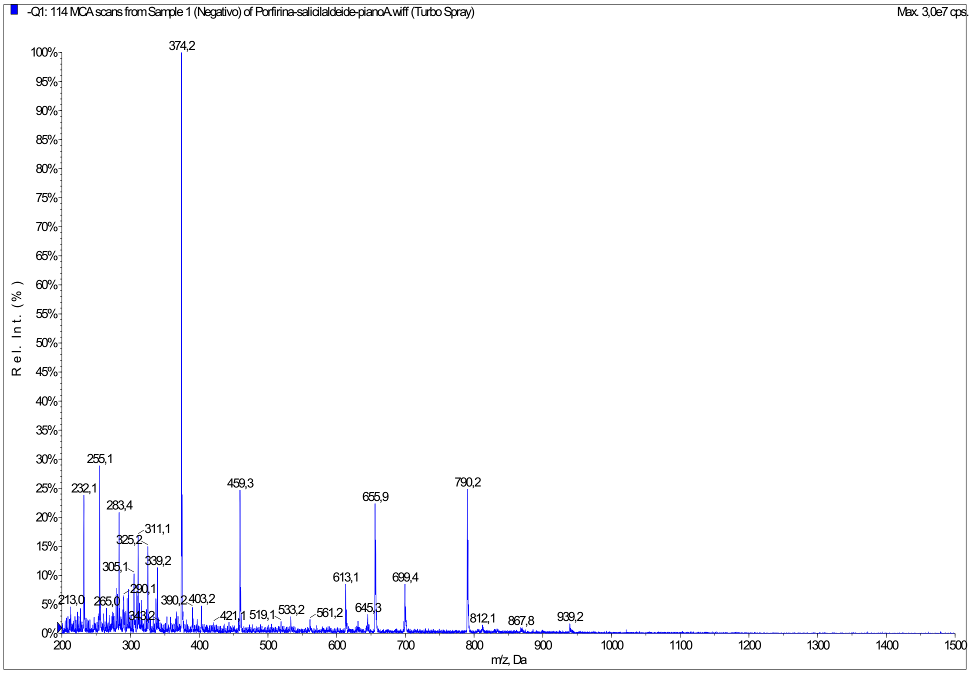

Supplementary Figure 14. ESI-MS compound **9**

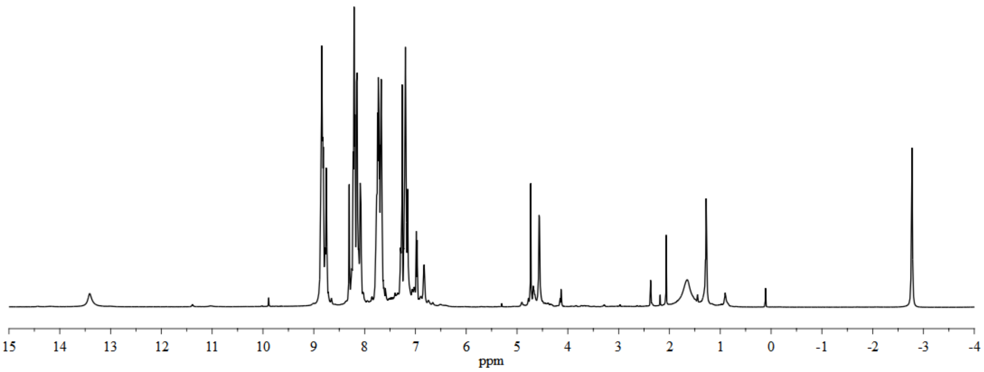

Supplementary Figure 15. ^{1}H-NMR of compound **10** in $CDCl_3$

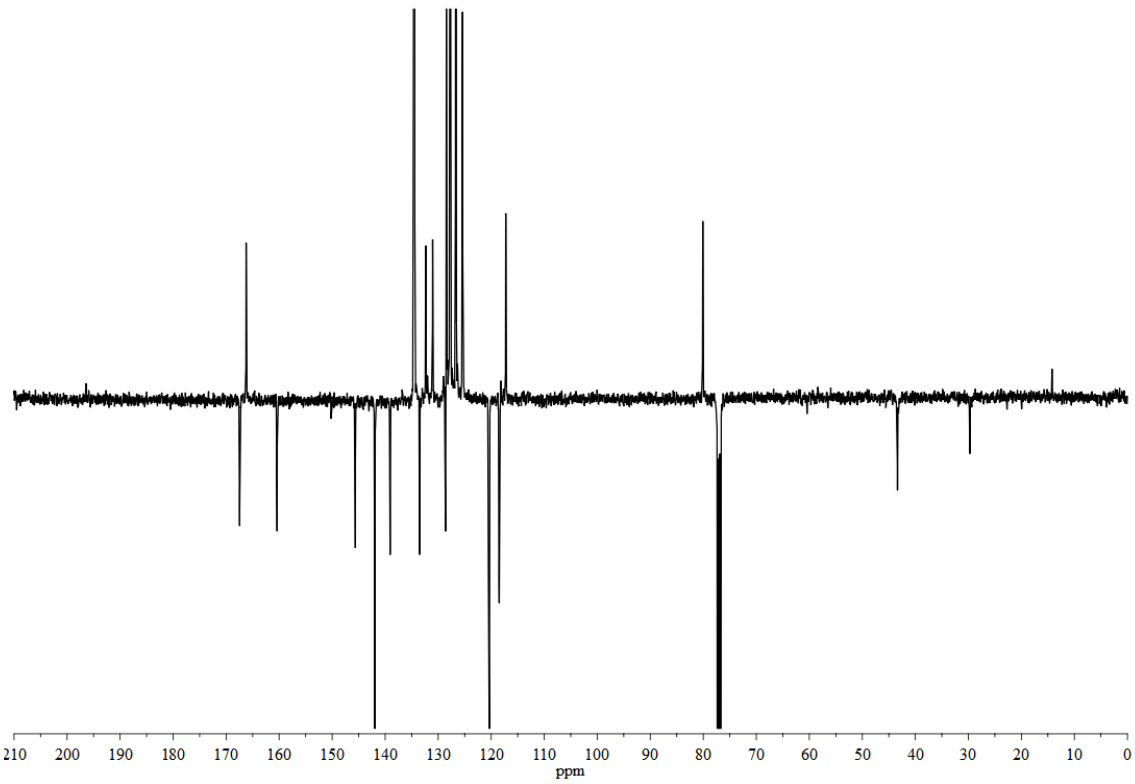

Supplementary Figure 16. APT of compound **10** in $CDCl_3$

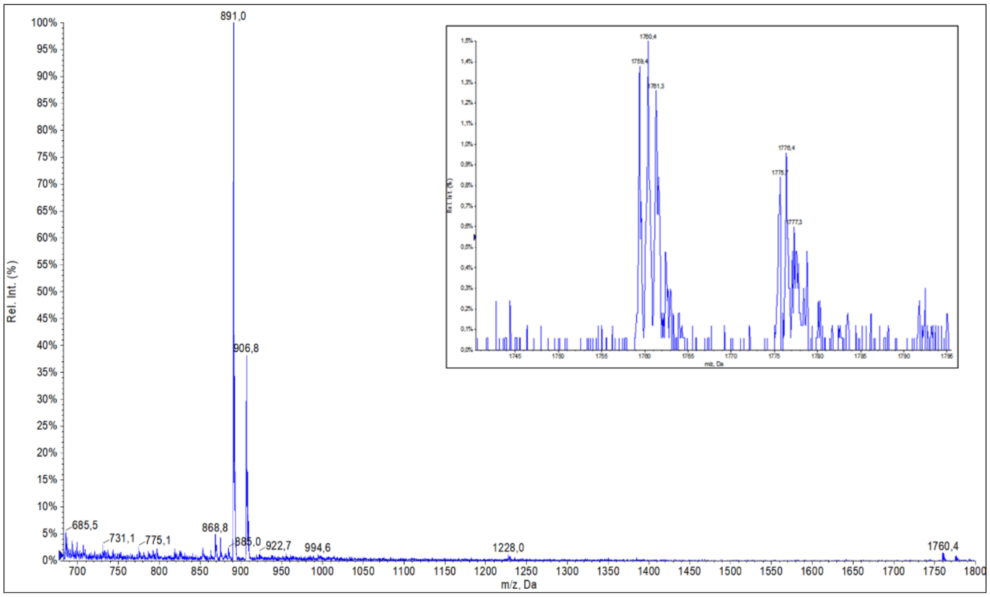

Supplementary Figure 17. ESI-MS of compound **10**
